# Supplementary material for: Cell-type-specific circadian and light-responsive transcriptional dynamics in adult Drosophila neurons
Source: Proc Natl Acad Sci U S A. 2026 Sep 8;123(37):e2619143123. doi: 10.1073/pnas.2619143123 (PMC13578309; doi:10.1073/pnas.2619143123)
Supplement: Supplementary file 1 [file pnas.2619143123.sapp.pdf]

PNAS

Supporting Information for

Cell-type-specific circadian and light-responsive transcriptional dynamics in adult Drosophila neurons

Gillian Berglund[1,2,3], Pranav Ojha[1,2], Maria Ivanova[1,2], Melina Pérez Torres[1,2,4], Michael Rosbash[1,2]*

1. Howard Hughes Medical Institute, Dept. of Biology, Brandeis University, Waltham, MA 02454
2. Department of Biology, Brandeis University, Waltham MA, 02454
3. Current Address: Department of Molecular Biosciences, Northwestern University, Evanston, IL, 60208
4. Current Address: Cold Spring Harbor Laboratory, Cold Spring Harbor, NY 11724

*Correspondence – rosbash@brandeis.edu

This PDF file includes:

Figures S1 to S6
Tables S1

Figures

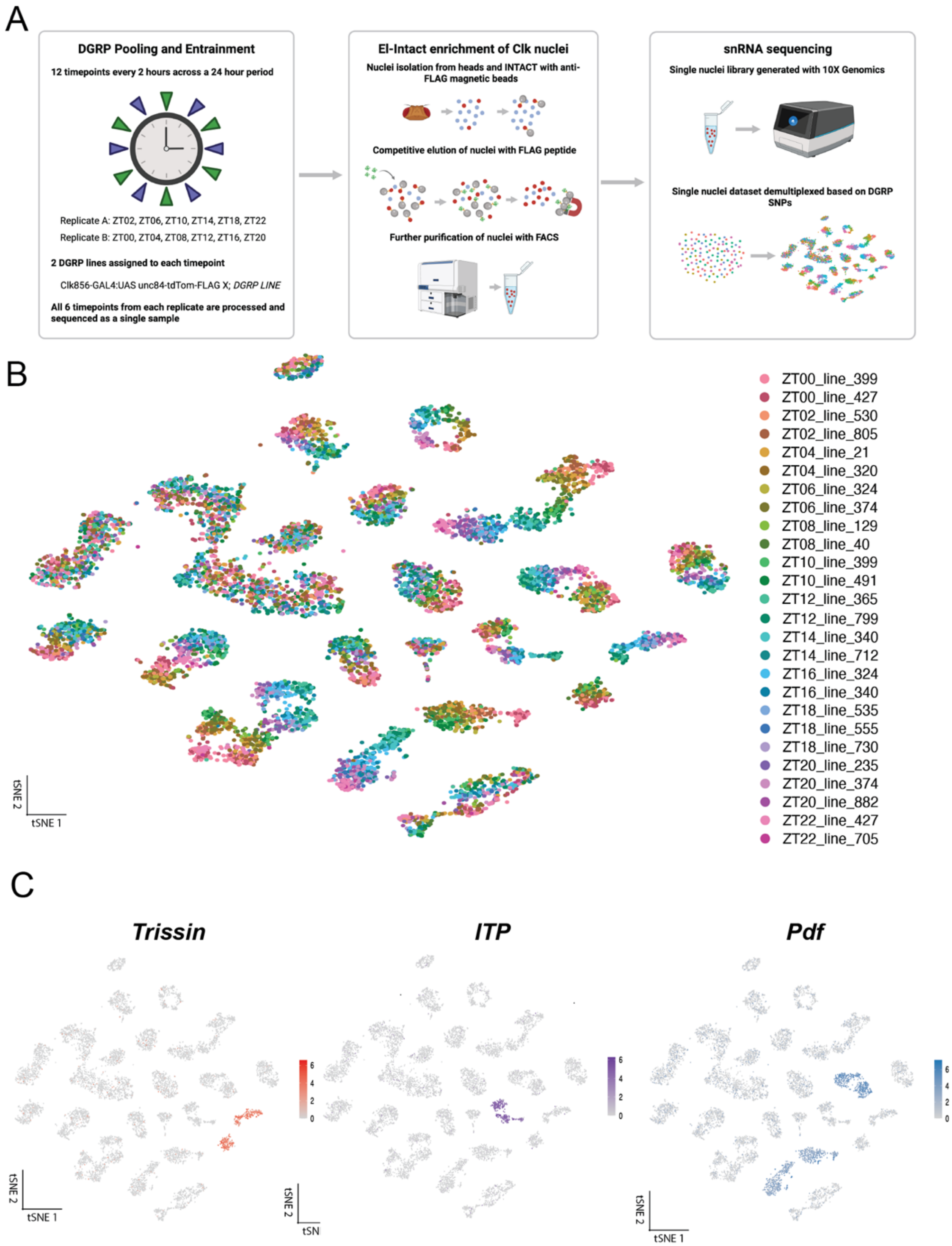
A
DGRP Pooling and Entrainment
12 timepoints every 2 hours across a 24 hour period
Replicate A: ZT02, ZT06, ZT10, ZT14, ZT18, ZT22
Replicate B: ZT00, ZT04, ZT08, ZT12, ZT16, ZT20
2 DGRP lines assigned to each timepoint
Clk856-GAL4:UAS unc84-tdTom-FLAG X; DGRP LINE
All 6 timepoints from each replicate are processed and sequenced as a single sample
El-Intact enrichment of Clk nuclei
Nuclei isolation from heads and INTACT with anti-FLAG magnetic beads
Competitive elution of nuclei with FLAG peptide
Further purification of nuclei with FACS
snRNA sequencing
Single nuclei library generated with 10X Genomics
Single nuclei dataset demultiplexed based on DGRP SNPs
B
ZT00_line_399
ZT00_line_427
ZT02_line_530
ZT02_line_805
ZT04_line_21
ZT04_line_320
ZT06_line_324
ZT06_line_374
ZT08_line_129
ZT08_line_40
ZT10_line_399
ZT10_line_491
ZT12_line_365
ZT12_line_799
ZT14_line_340
ZT14_line_712
ZT16_line_324
ZT16_line_340
ZT18_line_535
ZT18_line_555
ZT18_line_730
ZT20_line_235
ZT20_line_374
ZT20_line_882
ZT22_line_427
ZT22_line_705
tSNE 2
tSNE 1
C
Trissin
ITP
Pdf

Figure S1: Methods and results of single-nuclei RNA sequencing of *Drosophila* clock neurons. A) Schematic of experimental design for generating and processing single nuclei datasets using El-INTACT and pooled genetic multiplexing design. Flies with td-tomato-FLAG tagged labeled circadian neurons were collected across 12 time points, 2 hours apart split into two replicates of 6 time points. Td-tomato-tagged circadian nuclei were isolated using anti-FLAG magnetic beads, competitively eluted with FLAG peptide and purified with FACS. Single-nuclei RNA sequencing libraries were generated using single-cell 3' RNA seq (10X Genomics). The schematic of the workflow was created using BioRender (https://biorender.com). B) T-SNE plot showing each unique genotype and timepoint combination colored separately. C) T-SNE plots showing key lateral neuron marker genes expression: *Trissin*, *ITP*, and *Pdf* (color bar, TP10K).

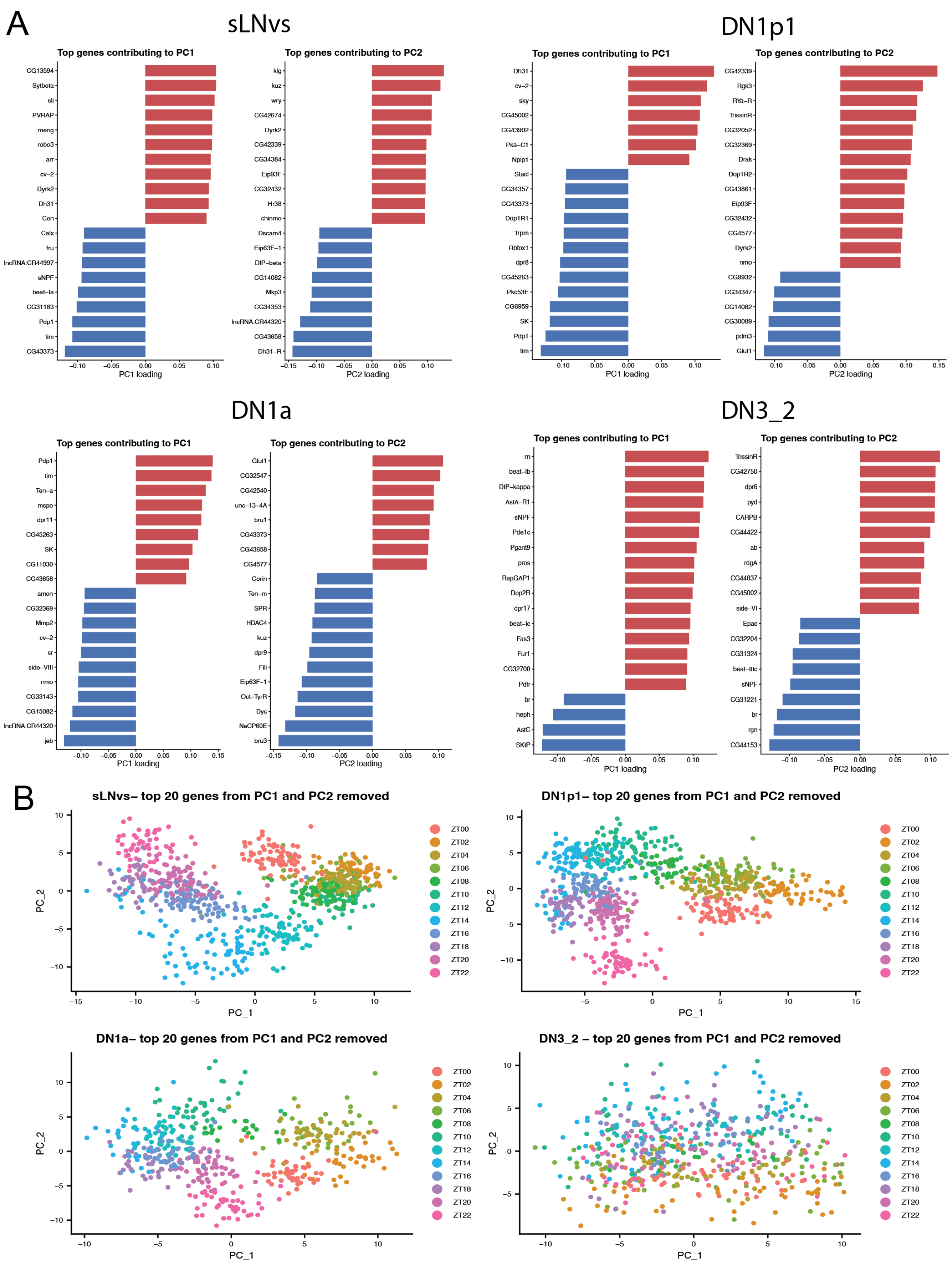

Fig. S2: Top genes driving time-of-day-dependent transcriptional continuum within cell types. A) Top 20 genes (x-axis) driving the variation captured by PC1 and PC2, and their relative loadings (y -axis) for a subset of circadian cell types (sLNvs, DN1p1,

DN1a, DN3_2). **B)** Principal component analysis (PCA) of subclustered sLNvs, DN1p1s, DN1a and DN3_2 cell types with the 40 genes shown in A removed.

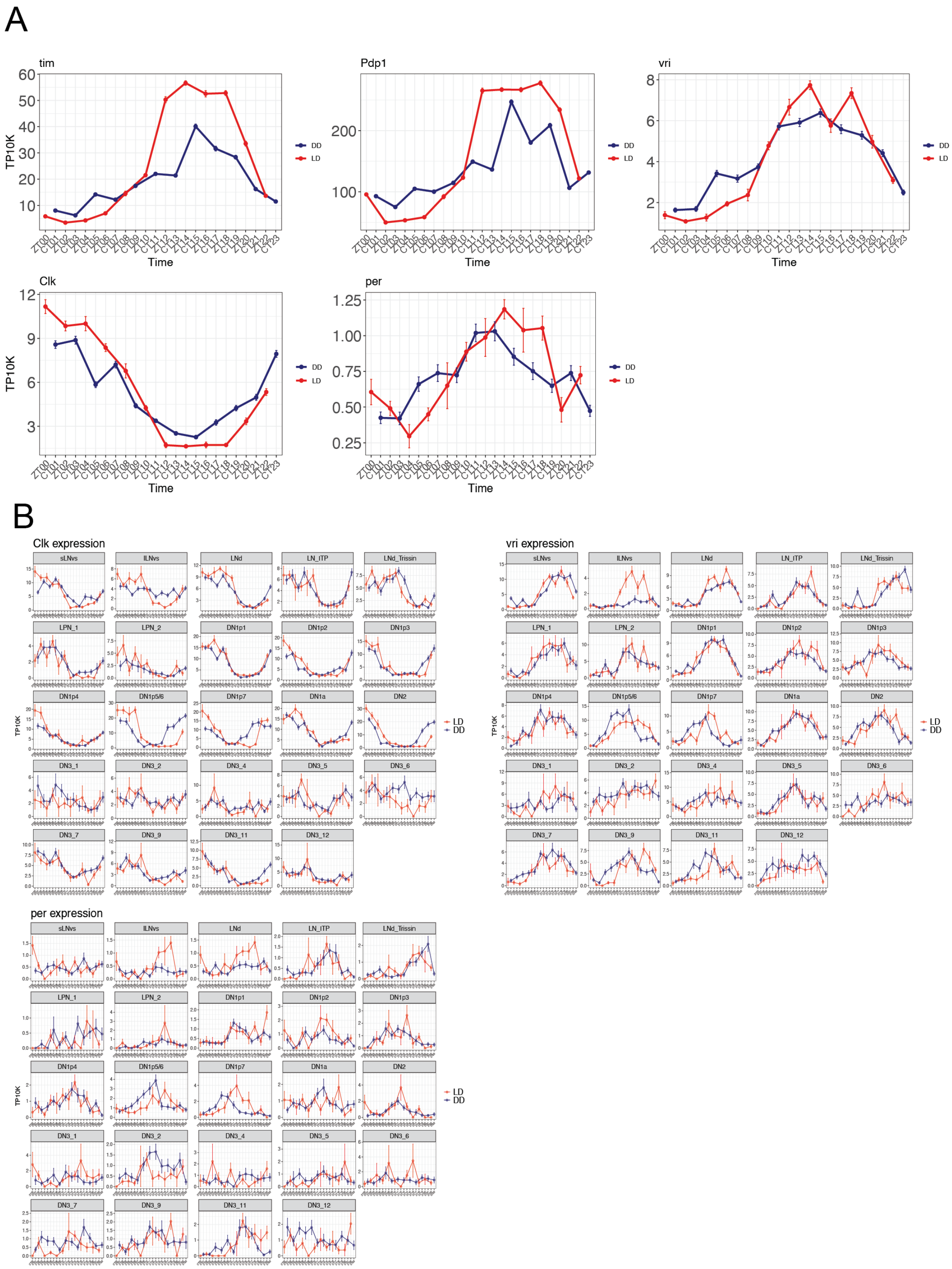

Fig. S3: Expression of core clock genes in 12 time point snRNA-seq. A) Pseudobulk expression of core clock genes, *tim*, *Pdp1*, *vri*, *Clk*, *per*. B) The mean *Clk*, *vri*, and *per* gene expression throughout the day in light: dark (LD) and constant darkness (DD)

conditions is graphed for each cluster. X axis indicates the time points in LD and DD and Y axes are scaled independently for cluster. Error bars represent mean ± SEM. Red and blue lines indicate the gene expression in LD and DD conditions, respectively.

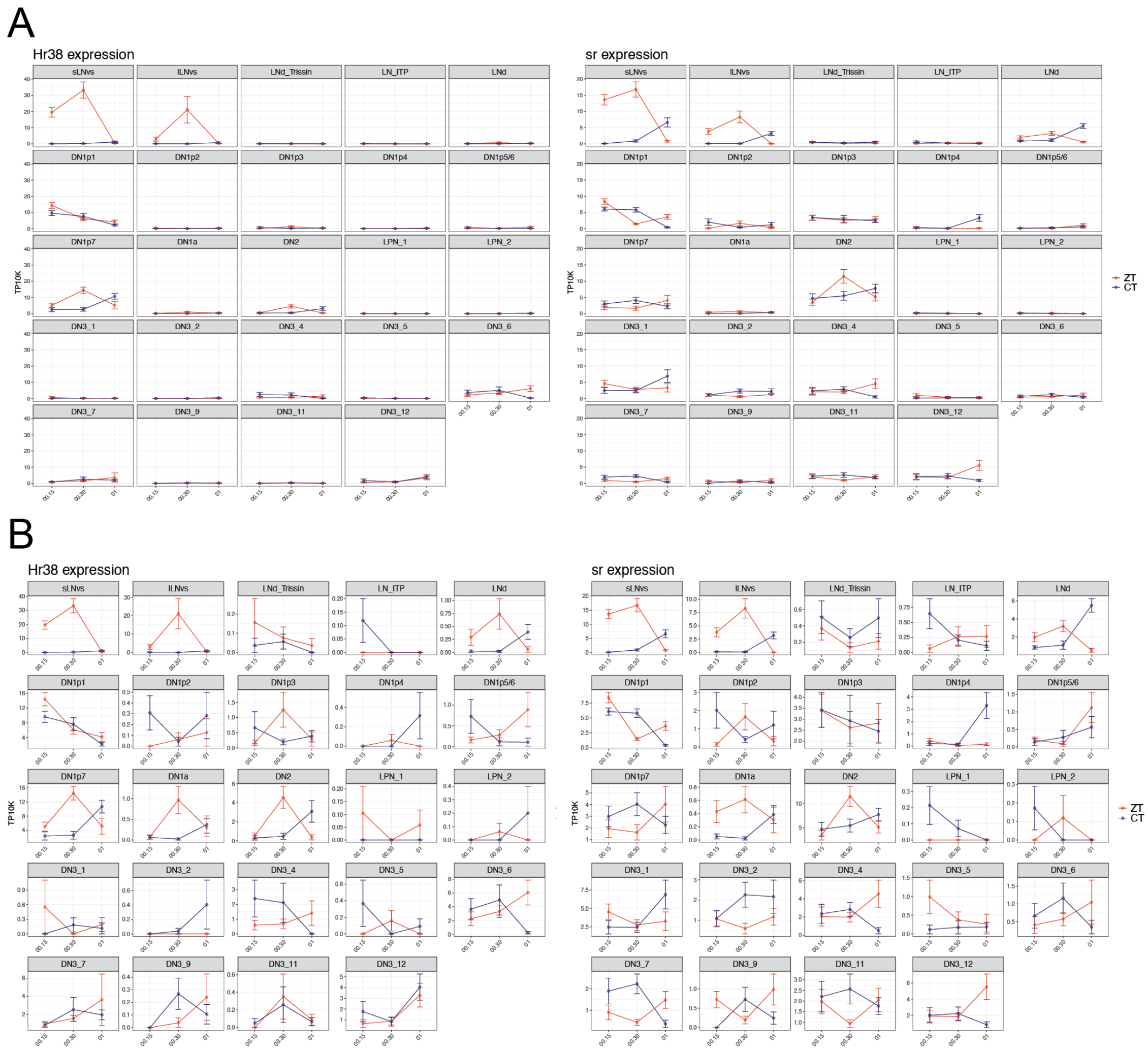

Fig. S4: Hr38 and Sr show characteristic ARG expression with time course around lights on.

A) The mean *Hr38* (left) and sr (right) expression following lights on at ZT00 in ZT conditions (red) or without light in the first day of DD (CT, blue). X axis indicates the time points following ZT00. Error bars represent mean ± SEM. B) *Hr38* and *Sr* expression of the same time course but with an independently scaled Y axis for each cluster.

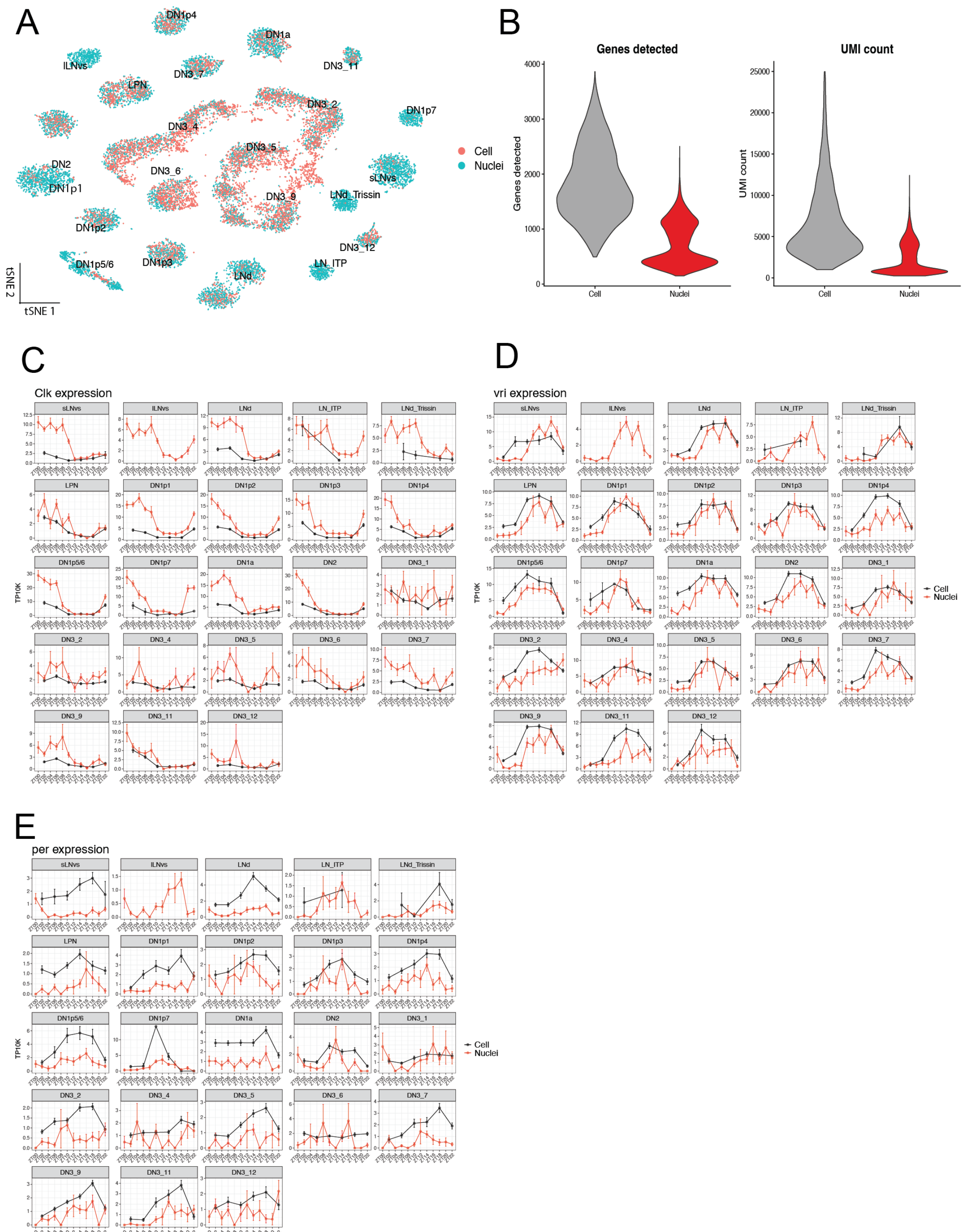

Fig. S5: Expression of core clock genes in scRNA-seq and snRNA-seq. A) t-SNE of integrated scRNA-seq and snRNA-seq LD datasets, with clusters colored by dataset. B) Number genes detected and UMIs per cell in datasets used in analysis. The average reads per nuclei was 25,665 and 48,432 for cells (C-E). The mean *Clk* (C), *Vri* (D), and *per* (E)

expression throughout the day in snRNA-seq and scRNA-seq datasets graphed for each cluster. X axis indicates the time points in LD and Y axes are scaled independently for cluster. Error bars represent mean ± SEM. Red and black lines indicate the gene expression in nuclei and cells, respectively.

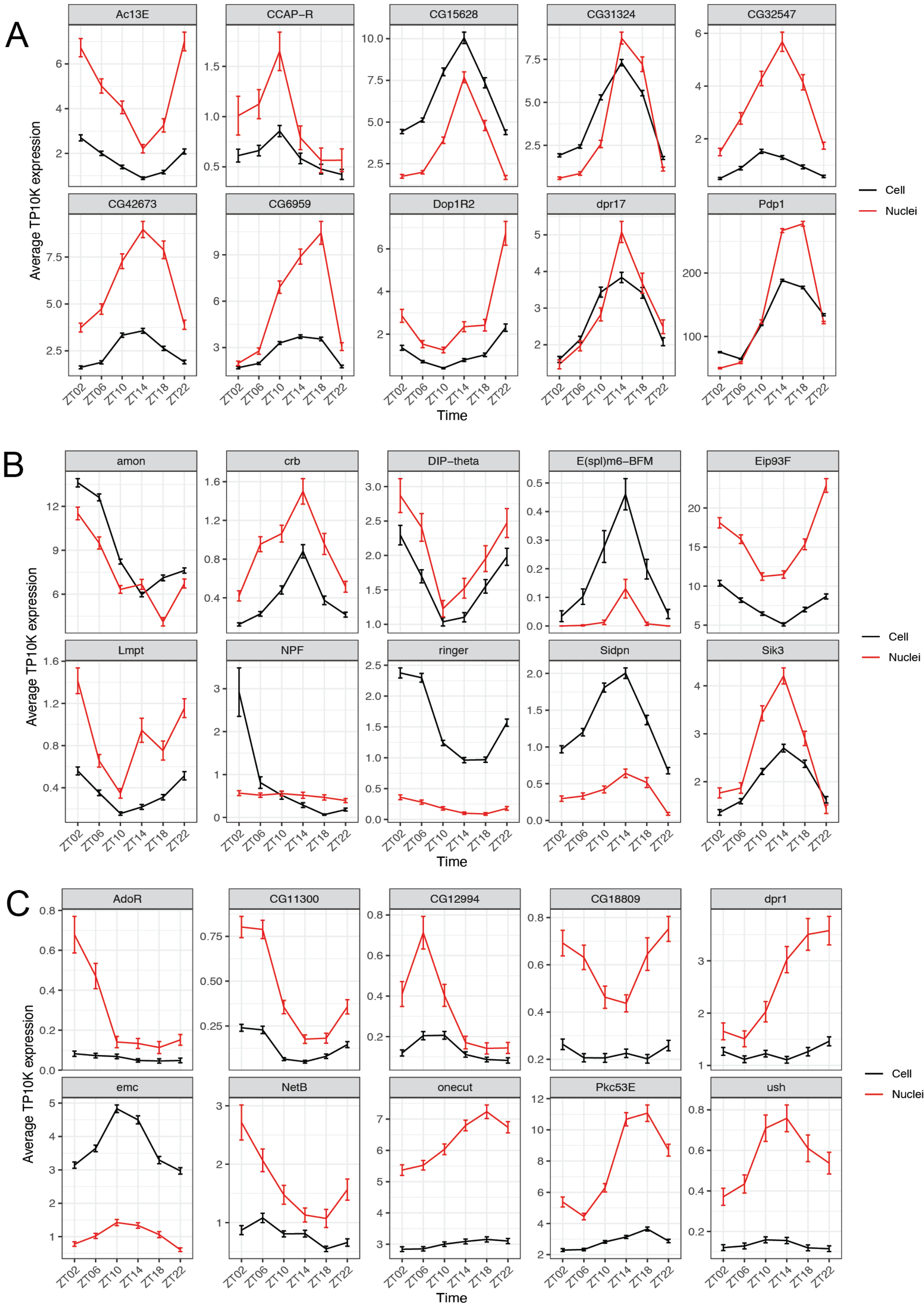

Fig. S6: Pseudobulk expression of additional cycling transcripts in either cells, nuclei or both. A) Random selection of 10 genes from list of genes identified as cycling

in both cell and nuclei dataset. B) Random selection of 10 genes from list of genes identified as uniquely cycling in cell dataset. C) Random selection of 10 genes from list of genes identified as uniquely cycling in nuclei dataset. Red and black lines indicate the gene expression in nuclei and cells, respectively. Y axes are scaled independently for each gene and error bars represent mean ± SEM.

Tables

Table S1: Summary of RNA sequencing datasets and quality control metrics.

Dataset:	Reads:	Number of cells/nuclei detected	Sequence saturation: (average reads per cell/nuclei)
Nuclei LD 1	219,284,138	9,354	23,549
Nuclei LD 2	273,239,400	9,835	27,782
Nuclei DD 1	547,481,146	9,690	56,500
Nuclei DD 2	535,828,993	11,507	46,565
Cell LD 1	475,351,430	21,379	22,235
Cell LD 2	492,186,293	6,595	74,630
